# HYPERTENSION CONTROL AND ISCHEMIC STROKE RISK: REAL-WORLD DATA IN HISPANIC MEDICARE BENEFICIARIES IN SOUTH FLORIDA

**DOI:** 10.1093/geroni/igae098.2457

**Published:** 2024-12-31

**Authors:** Paulo Chaves, Maureen Desoria, Leslye Roque, Rafael Mas

**Affiliations:** Florida International University, Miami, Florida, United States; Leon Medical Centers, LLC, Miami, Florida, United States; Leon Medical Centers, LLC, Miami, Florida, United States; Leon Medical Centers, LLC, Miami, Florida, United States

## Abstract

The extent to which the magnitude of stroke risk reduction associated with blood pressure (BP) control in highly controlled trial research settings is translated to real world clinical settings remains to be better characterized, particularly in understudied populations. We examined the odds of hospitalization for acute stroke as a function of BP control in the previous year in Medicare Beneficiaries of Hispanic origin in south Florida. We analyzed de-identified electronic health records (2015-2019) from outpatients ≥65 years old receiving care at Leon Medical Centers (LMC), a major integrated healthcare services provider to Medicare and Medicare/Medicaid dually eligible (DE) Beneficiaries in Miami-Dade County, FL. In each year, within-person average systolic (SBP)< 140 mmHg and diastolic (DBP)< 90 mmHg were used to define controlled BP. Acute ischemic strokes were identified from hospital claims (ICD-CM-10 codes). Pooled longitudinal logistic regression with 1-year lagged time (n=36,519) with adjustment for age, sex, and DE status. Mean age (76.1±7.2 to 77.5±7.2), proportion of Hispanics (>90%), and annual BP control rates (>90%) remained constant throughout 2015-2019. Patients with controlled BP in a specific year had 39% lower (OR.61; 95%CI: 0.51, 0.73) odds of hospitalization for acute stroke in the subsequent year than those with uncontrolled BP. Neither sex, nor DE status modified the BP control–stroke risk relationship. In a large healthcare system in south Florida, we observed very high BP control levels, and, in line with published trial data, documented a substantially lower risk of ischemic stroke 1 year later in both Medicare and DE patients of Hispanic origin.

